# Supplementary material for: End Sequence Analysis Toolkit (ESAT) expands the extractable information from single-cell RNA-seq data
Source: Genome Res. 2016 Oct;26(10):1397–410. doi: 10.1101/gr.207902.116 (PMC5052061; doi:10.1101/gr.207902.116)
Supplement: Supplemental Material [file supp_gr.207902.116_Supp_Materials.tar.gz › ESAT-esat_v0.1/doc/html/classjaligner_1_1util_1_1_sequence_parser.html]

ESAT: jaligner.util.SequenceParser Class Reference


ESAT


- Main Page
- Related Pages
- Packages
- Classes
- Files

- Class List
- Class Index
- Class Hierarchy
- Class Members

All Classes Namespaces Files Functions Variables Enumerator Pages

- jaligner
- util
- SequenceParser

Static Public Member Functions |
List of all members

jaligner.util.SequenceParser Class Reference

|  |  |
| --- | --- |
| Static Public Member Functions | |
| static Sequence | parse (String sequence) throws SequenceParserException |
|  | |
| static Sequence | parse (File file) throws SequenceParserException |
|  | |

## Detailed Description

SequenceParser to sequences from different formats.   
Currently the supported formats are:

- Plain sequence
- Sequence

Author
:   Ahmed Moustafa (ahmed.nosp@m.@use.nosp@m.rs.sf.nosp@m..net)

## Member Function Documentation

|  |  |  |  |  |  |
| --- | --- | --- | --- | --- | --- |
| static Sequence jaligner.util.SequenceParser.parse | ( | String | *sequence* | ) | throws SequenceParserException |

static

Returns a parsed Sequence from a string.

Parameters
:   |  |  |
    | --- | --- |
    | sequence | string to parse |

Returns
:   parsed sequence

Exceptions
:   |  |  |
    | --- | --- |
    | SequenceParserException |  |

See also
:   Sequence

|  |  |  |  |  |  |
| --- | --- | --- | --- | --- | --- |
| static Sequence jaligner.util.SequenceParser.parse | ( | File | *file* | ) | throws SequenceParserException |

static

Returns a Sequence parsed and loaded from a file

Parameters
:   |  |  |
    | --- | --- |
    | file | to parse |

Returns
:   parsed sequence

Exceptions
:   |  |  |
    | --- | --- |
    | SequenceParserException |  |

See also
:   Sequence

---

The documentation for this class was generated from the following file:

- java/jaligner/util/SequenceParser.java


---

Generated on Fri Aug 15 2014 15:11:06 for ESAT by  

 1.8.7
